# Pupil dilation but not microsaccade rate robustly reveals decision formation

**DOI:** 10.1038/s41598-018-31551-x

**Published:** 2018-09-03

**Authors:** Christoph Strauch, Lukas Greiter, Anke Huckauf

**Affiliations:** Ulm University, General Psychology, Ulm, 89081 Germany

## Abstract

Pupil dilation, an indicator of arousal that is generally regarded as unspecific, amongst others reflects decision formation and reveals choice. Employing letter selection in a Go/NoGo task, we show that choice can robustly be predicted by the pupillary signal, even under the presence of strong interfering factors such as changes in brightness or motor execution. In addition, a larger difference in pupil dilation between target and distractor conditions for NoGo compared to Go was demonstrated, underlining the particular appropriateness of the paradigm for decision research. Incorporating microsaccades, a variable that is suggested to covary with pupil diameter, we show that decision formation can only be observed in pupil diameter. However, microsaccade rate and pupil size covaried for motor execution and both reflected choice after key press with smaller effect size for microsaccade rate. We argue that combining pupil dilation and microsaccade rate may help dissociating decision-related changes in pupil diameter from interfering factors. Considering the interlinked main neural correlates of pupil dilation and microsaccade generation, these findings point to a selective role of locus coeruleus compared to superior colliculus in decision formation.

## Introduction

Variations in pupil diameter, at constant brightness and viewing distance, are a popular but unspecific indicator of cognitive^1^ and affective processing^2–5^. Pupil diameter reportedly tracks the activity of noradrenergic locus coeruleus (LC)^6–11^. The hereby modulated arousal level might thus contribute to the covariation of various mental processes and pupil diameter. Decision making has been investigated by means of pupillometry in several tasks^5,7,9,12–18^. It was found that pupils dilate stronger when deciding for “yes” than for “no” in a signal detection task, likely reflecting the accumulation of information until a response criterion is reached^18,19^. Furthermore, manual responses could be predicted, suggesting that pupil dilation not only reflects choice, but also the foregoing decision formation^12,19^. Given the low specificity of pupil diameter, it remains doubtable whether decision formation and choice can still be identified in the presence of several other factors. This is especially the case in the presence of factors producing considerably larger effects like, for example, brightness changes. Two approaches might serve for compensating the missing specificity; firstly, one might disentangle interfering effects, or, secondly, one might add a further variable. Both approaches were pursued by examining pupil responses in a decision task including strong interference factors such as a sudden change in brightness, a key press, a tone, and four directions of incoming saccades while simultaneously tracking microsaccade rate.

Pupil size and microsaccade rate respond to similar psychological phenomena even when stimulus material is kept constant regarding its sensory and spatial characteristics: Two recent investigations combined pupil dilation and microsaccade rate in the context of visual search and found an overlap in results between the two variables^20,21^. Similarly, in an oddball paradigm, it is claimed that microsaccade rate might change differentially for odd stimuli in comparison to other stimuli^22,23^, while it has been reported that pupil dilation is also affected by the oddness of a stimulus^8^. Another psychological phenomenon that is linked to changes in pupil diameter and microsaccade rate is cognitive load^24,25^. Here, higher workload is associated with larger pupils and a lower rate of microsaccades^25^. Moreover, also neural correlates of pupil dilation, locus coeruleus (LC), and of microsaccade rate, superior colliculus (SC), are reported to be closely connected^8,18,26^. Correspondingly, in animal experiments, microstimulation in the SC and in the LC is associated with pupil dilations^6,26^. Interestingly, despite finding a close correlation of activity in brainstem nuclei, also including LC and SC, only activity in LC, but not in SC predicted changes in decision bias during evidence accumulation for decision making^18^. Therefore, during decision formation, pupil diameter but not microsaccade rate might be modulated. However, after deciding, effects could be visible in both pupil dilation and microsaccade rate.

Pupil diameter and microsaccade rate are variables that have been separately employed in hundreds of investigations into psychological phenomena that are partially similar. However, both dependent variables are hardly conjointly investigated for several psychological factors; a gap in existing research that we aim to close. Besides this methodological contribution, we deepen the fundamental understanding of binary decision making and investigate the robustness of the effects of choice on pupil diameter in the presence of strong interfering variables. By combining these two ideas, application scenarios for pupil diameter and microsaccade rate in human-computer interaction could emerge, e.g. prediction of user intention^27^.

## Results

**Figure 1:**
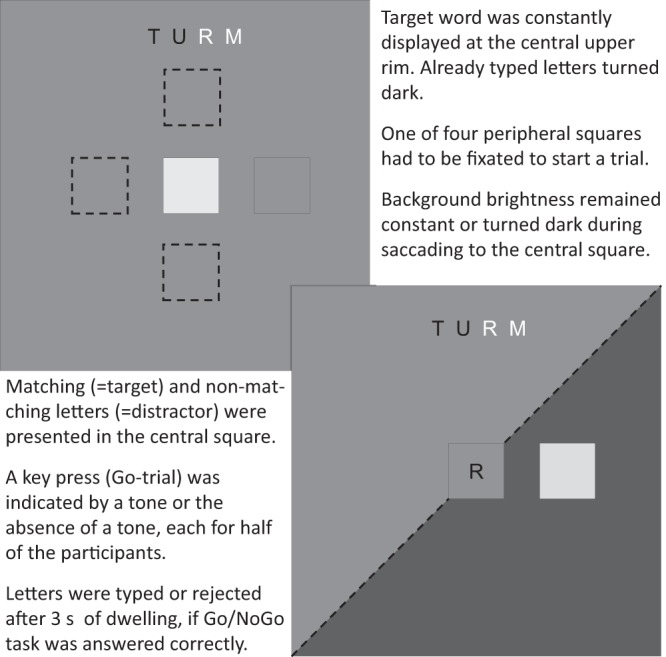
Events in the letter selection task.

Participants had to either select or reject letters during fixating a central square (Fig. 1). During a trial, participants had to firstly fixate an outer square and then saccade to the center. Upon fixation of the central square, a letter appeared. The letter either matched (=target) or mismatched (=distractor) the next letter in the word to be written and presented above. This operationalization for choice carries the advantage of physically identical stimulus material that only differs in the assigned meaning. In half of the trials, the screen abruptly turned dark while saccading to the central square. At the same time, for half of trials, a tone either prompted participants to press (=Go) or not to press a key (=NoGo) to indicate whether the presented letter was correct or not. Trials were presented in random order, distractors and targets were equally likely to appear.

### Pupil diameter reacts to many variables, but robustly reveals decision formation and choice

**Figure 2:**
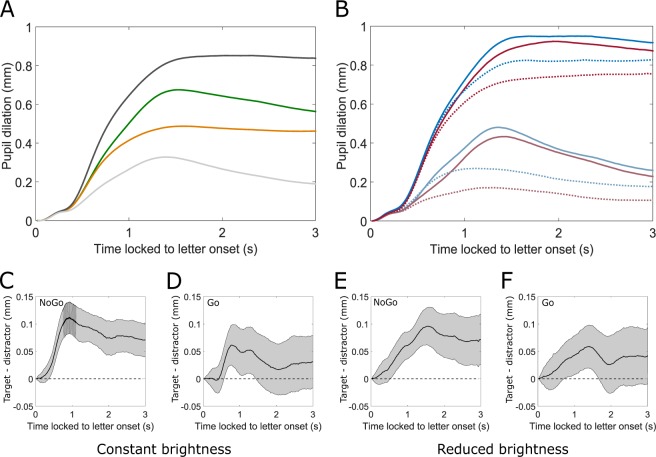
Stimulus-locked pupil data. (**A**) Changes in pupil diameter for constant brightness condition (light gray), reduced brightness condition (black), Go- (green), and NoGo-condition (orange). (**B**) Pupil diameter for target-trials (blue) and distractor-trials (red) show that pupil always responded stronger when targets were present. This effect could be monitored under both brightness conditions (upper four lines: reduced brightness) and irrespective of the Go/NoGo task (Go: solid lines, NoGo: dotted lines). (**C**–**F**) Differences between target and distractor together with functional confidence intervals. If zero is not contained in the CI, changes are significantly different to a FDR-corrected *α* = 0.05. All conditions showed clearly significant differences between target- and distractor-trials. (**C**) Constant brightness, NoGo. (**D**) Constant brightness, Go. (**E**) Reduced brightness, NoGo. (**F**) Reduced brightness, Go.

Pupil data were blink-filtered and subsequently baseline-corrected. Individual trials were averaged to condition-average signal courses within subjects. Differences in signal courses between conditions were calculated for each subject. These curves were then aggregated to grand mean dynamics. Confidence intervals were calculated over these grand mean dynamics for each data point (functional confidence intervals) to a False-Discovery Rate (FDR) corrected *α* = 0.05. For differences between conditions, a statistically significant effect is indicated whenever zero is not contained in the confidence interval. As Fig. 2A shows, there was an expected strong influence of the investigated factors (constant/reduced brightness, with/without key press). However, the direction of the incoming saccade did not lead to significantly different pupil courses, apart from the saccade going downwards. Still, this might be convoluted due to the target-word that has been written above (Fig. 1). In Fig. 2B, the average pupil dilation following letter fixation is displayed for target and distractor conditions in Go- and NoGo-trials, as well as in the two background brightness conditions respectively. It is discernible that target letters led to a descriptively larger pupil dilation compared to distractor letters in all corresponding configurations of brightness and key press. This difference is illustrated for all four conditions respectively (Fig. 2C NoGo, constant brightness, Fig. 2D Go, constant brightness, Fig. 2E, NoGo, reduced brightness, Fig. 2F Go, reduced brightness). In all conditions, functional confidence intervals revealed a significantly larger pupil dilation for targets than for distractors, albeit for slightly varying durations and magnitudes. This descriptive pattern was found for most, but not all subjects (Supplementary Fig. S1). While the presence of a tone led to a significantly larger pupil than no tone for about 300 ms, no interaction with choice could be observed (Supplementary Fig. S2).

### Modeling pupil data suggests fast and robust differentiation of target and distractor

**Figure 3:**
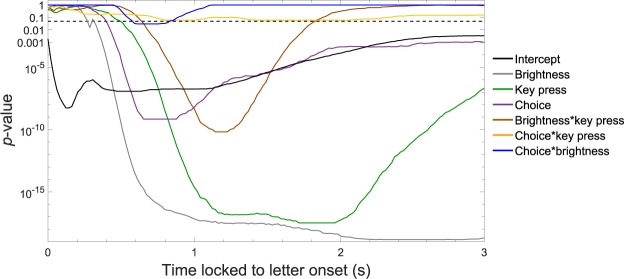
Functional *p*-values (FDR-corrected) of a functional linear mixed model (LMM) wih the factors brightness, key press, choice, and their interactions. The dashed line indicates *p* = 0.05. Functional *p*-values reveal highly significant effects for brightness, key press, choice, and the interaction of brightness and key press, but only a short significant interaction for brightness and choice, and no significant effect for the interaction of choice and key press.

Pupil courses suggest a reliable differentiation between target and distractor even faster than 1.5 s. In order to account for the multifactorial design, a linear mixed model (LMM) was calculated for 0.5 s, 0.75 s, 1 s, 1.25 s, and 1.5 s after letter onset. Hereby, main effects and their interactions on pupil diameter can be retrieved. Table 1 depicts the best fitting linear mixed model. The predictors revealed differential and partially transient influence on pupil diameter. Explained variance increases over time and peaks at 1.5 s, with a *R*^2^ of 0.622. Furthermore, a functional LMM was calculated for key press, brightness, and choice for all data points of the three seconds of stimulus presentation, which is visualized in Fig. 3. As can be seen, the intercept, brightness, key press, choice, and the interaction between key press and brightness reveal highly significant differences in pupil diameter. At the same time, the interactions for brightness and choice, as well as choice and key press are mostly not significant. Of course, here partially very low *p*-values should not be over interpreted, but rather treated as being beyond a threshold of *p* = 0.001. However, they also give an impression of the effect sizes at hand.

**Table 1:** Unstandardized predictors of a linear mixed model (LMM) for pupil dilation for five time intervals after letter onset.

Predictors	0.5 s	0.75 s	1 s	1.25 s	1.5 s
Intercept	0.041	0.013	0.010	0.002	−0.034
Brightness	**0.113**	**0.337**	**0.467**	**0.546**	**0.628**
Key press	0.012	**0.062**	**0.150**	**0.239**	**0.219**
Saccade direction	0.58	**0.136**	**0.166**	**0.183**	**0.200**
Choice	0.019	**0.070**	**0.092**	**0.093**	**0.086**
Tone	0.005	**0.039**	**0.044**	**0.057**	**0.032**
Key press*choice	−0.011	−0.022	**−0.039**	−0.035	**−0.055**
Brightness*choice	−0.004	**−0.041**	**−0.083**	**−0.118**	−0.042
Brightness*direction	**−0.031**	**−0.052**	**−0.062**	**−0.059**	−0.033
R^2^	0.314	0.503	0.574	0.604	0.622

Bold numbers indicate statistical significance of predictors to *α* = 0.05 level (Bonferroni corrected). Only downwards saccades differed from all other directions for the factor saccade direction.

### Pupil dilation predicts which key will be pressed

**Figure 4:**
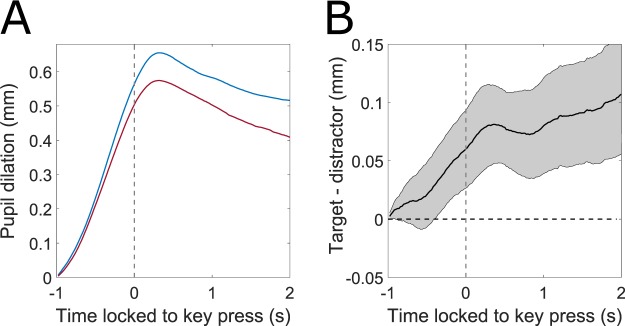
(**A**) Response-locked pupil dilation (Go-trials; blue: target, red: distractor). (**B**) Differences between target and distractor for the same trials as in A in conjunction with functional confidence intervals. If zero is not contained in the CI, changes are significantly different to FDR-corrected *α* = 0.05. Pupil diameter in target and distractor conditions were significantly different from 400 ms prior to key press.

In Go-trials, subjects needed an average of *M* = 1.07 s (*SD* = 0.15 s; average fastest reaction time per participant *M* = 0.68 s, *SD* = 0.06 s) to press the correct key. Only four trials were answered faster than 0.5 s across all subjects. Comparing average reaction times in Go-trials with predictors in Fig. 3 suggests that pupil diameter revealed earlier than the key press whether a target or a distractor letter was presented. Figure 4A illustrates pupil diameter changes response-locked to the key press for Go-trials in target- and distractor-conditions. Pupils dilated stronger for target- than distractor-letters, even before subjects were capable of responding overtly. In Fig. 4B, the functional confidence intervals indicate a significant difference between target and distractor starting from 400 ms prior to key press. The magnitude of the difference for these Go-trials was approximately of similar magnitude as for NoGo-trials (Fig. 2C,E). Reaction times for target (*M* = 1.08 s) and distractor trials (*M* = 1.05 s) significantly differed as shown by a paired samples t-test (*p* = 0.005, *t*(29) = 2.99). Still, the difference of 30 ms in reaction time would have been too small to fully account for the difference of 400 ms between target and distractor conditions.

### Microsaccade rate does not predict choice but indicates a key press

**Figure 5:**
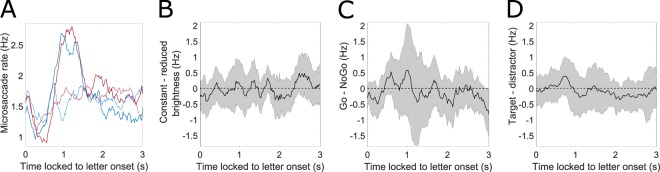
Stimulus-locked microsaccade data. (**A**) Microsaccade rate over time for target (blue), distractor (red), for Go-(solid lines) and NoGo-trials (dotted lines) respectively. (**B**–**D**) Differences in microsaccade rate with functional confidence intervals. If zero is not contained in the CI, changes are significantly different to FDR-corrected *α* = 0.05. Microsaccade rate did not differ significantly between target and distractor, brightness conditions, and between Go and NoGo.

**Figure 6:**
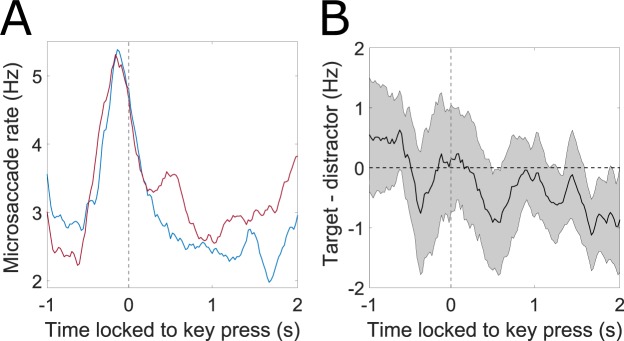
(**A**) Response-locked microsaccade rates (Go-trials; blue: target, red: distractor). (**B**) Differences between target and distractor for the same trials as in A in conjunction with functional confidence intervals. If zero is not contained in the CI, changes are significantly different to FDR-corrected *α* = 0.05.

Microsaccades could be analyzed for 26 out of 30 subjects, due to a low sampling rate for the remaining 4 subjects. Microsaccade rate is illustrated in Fig. 5A for target and distractor in Go/NoGo conditions respectively. Typically for microsaccade rate, an initial suppression was found shortly after letter onset, followed by a transient higher probability of microsaccade occurrence^23,28–30^. Figure 5B shows that microsaccade rates did not differ between both levels of brightness. Also, the tone did not affect microsaccade rate significantly (Supplementary Fig. S3), still descriptive changes correspond to earlier significant findings^31^. In Fig. 5C, the difference in microsaccade rate between Go- and NoGo-trials is illustrated, while Go-trials are associated to a higher rate around the time of key press, this difference is not significant. Target and distractor did not differ overall (Fig. 5D). Figure 6A depicts microsaccade rate in Go-trials locked to the key press for target and distractor conditions. Although a clear increase in microsaccade rate can be found for Go-trials starting approximately 600 ms before the key press, there was no significant difference between target and distractor before key press. However, there was a significantly lower microsaccade rate observed for targets compared to distractors about 1.7 s after key press. Therefore, in contrast to the pupillary signal, while the microsaccade rate could predict pressing a key, it could not predict the choice of the key (target vs. distractor; Fig. 6B). Still, traces of choice were visible in the microsaccade rate with a lag when a key had been pressed. When aligning pupil responses to microsaccades that occurred during trials, no direct modulation of pupil diameter prior or post microsaccades was visible, but an overall positive trend, which likely reflects the increasing trend throughout trials (Supplementary Fig. S4).

## Discussion

By means of a letter selection task including Go/NoGo-trials, we investigated whether and how robust decision formation and the outcomes of a binary decision process can be revealed by analyzing pupil diameter. Further, we assessed microsaccade rate as a covariate in order to deepen the understanding of commonalities and differences in relation to pupil dilation.

Target and distractor letters could be distinguished by larger pupils for target compared to distractor letters in all conditions, that is, with constant and with changing background brightness, with key press and without. Pupil serves mainly as the aperture of the eye and therefore strongly reacts to changes in brightness, as demonstrated here, with latencies less than 250 ms^32^. Nevertheless, differences in pupil diameter between target and distractor conditions could be shown in both brightness conditions. Descriptively, the slopes for the choice effect differed between constant and reduced brightness (Fig. 2C–F). Accordingly, the functional LMM reveals a shortly significant interaction at this time (Fig. 3). The descriptively differential patterns in the pupil diameter courses between participants might be due to their idiosyncratic decision making strategies^19^ (Supplementary Fig. S1).

It has been shown that motor execution like pressing a key leads to a considerably dilated pupil even before executing the movement itself^5,9,33^. Accordingly, the key press had a clear effect on absolute pupil diameter and interacted with the effect of choice. Data replicated overall larger pupil dilations for trials with key press^13^. In contrast to existing research, the difference between target and distractor emerged stronger for NoGo-trials (Fig. 2 and Table 1). This finding might be explained by processes of inhibition^34^ or by a convolution of pupil data by the motor execution^33,35^ that took place at different times for the Go-trials. The latter assumption is supported by response-locked pupil data (Fig. 4).

Yet existing studies either involved visual target detection where no corresponding distractor stimuli were provided^20,21^, decisions were linked to a motor response in all trials^18,19^, or a key press was systematically necessary in one part of the experiment but never in another^13,20^. As to our knowledge, we are the first showing differential pupil sizes for target and distractor conditions, that only differed regarding their task-induced meaning, which had to be indicated after being prompted in half of trials. In contrast to yet existing data, we found larger decision effects for trials without key press than for trials with key press. Larger effects for key press conditions have repeatedly been explained by diminished involvement and motivation of participants for trials with no necessity for overt responses^13,20,36^. Other than in common experimental investigations on binary decision making without necessary key press so far, decisions in our everyday lives have a direct impact on ourselves with meaningful consequences. By shaping trials challengingly short, but long enough to monitor effects on pupil dilation, and making errors meaningful (i.e. incorrect words had to be “retyped” and prolonged the experiment), we sustained a high motivation and involvement of participants throughout all conditions. Hence, we conclude that our paradigm is particularly well suited for investigating decisional processes with eye-tracking.

In addition, pupil sizes have been supposed to respond to the direction of the incoming saccade^37^. All directions of incoming saccades led to similar pupil dilations with only downward saccades deviating. Modeling pupil diameter showed a significant interaction of saccade direction and brightness until 1.25 s (Table 1). We thus partially replicate findings on differing pupil diameter depending on the direction of the incoming saccade^37^. Since the starting point of the downward saccade also coincides with the position of the target word (and thus also with a slightly different brightness), we argue to treat this result carefully (Fig. 1). Hence, the effect of brightness and downward saccades on pupil diameter might be subject to future investigations. Moreover, pupil dilation is reflecting the orienting response^26,29^, which should partially account for the intercept in the linear mixed models depicted in Table 1. Given that the intercept was not significant for these data points, it can be assumed that the orienting response did not affect pupil diameter beyond the here defined statistically significant level.

For Go-trials, pupil diameter not only significantly predicted whether a key was pressed^33,35^, but also which response (select or reject) was given, as soon as 400 ms before key press. This replicates the finding of de Gee *et al*.^19^ of a larger pupil dilation for “yes”-choices, but in a different experimental paradigm that required higher cognitive functioning, i.e. deciding on a letter’s concordance to a target word. Given that simple reaction times, comprising stimulus detection and motor execution, take about 250 ms^38^, motor execution itself should not take substantially longer. Considering significantly different pupil sizes for target and distractor letters already from 400 ms before the key press in this experiment, our findings support de Gee *et al*.’s assumption of an indicative role of pupil diameter even during decision formation^19^. Taken together, this investigation highlights the potential of pupil diameter for investigating possibly preconscious decision formation as suggested before^19,39^. In this way, the predictive character of pupil diameter could be employed to reveal intention in real time, e.g. to enable fast and intuitive selection^27,40,41^.

Microsaccades, the fastest and largest of the fixational eye-movements^42^ were additionally investigated. Spontaneous microsaccade generation is described to range between 1 Hz and 2 Hz^23,43^. Similar to changes in pupil diameter, changes in microsaccade rate are linked to both top-down processes (i.e. cognition) and bottom-up processes (i.e. perception). In this experiment, microsaccade rates showed the typical course of suppression with a subsequent higher rate^23^. Here, the higher rate of microsaccade occurrence was found to be longer than in most other studies (Fig. 5A). The first decrease in microsaccade rate could signal both an ongoing decision process and an orienting response^29^. Microsaccade rate for target and distractor conditions, however, showed no differences overall (Fig. 5D). Still, when response-locking microsaccade rates, a significant effect of choice was monitored, with lower microsaccade rates for targets after about 1.7 s (Fig. 6B). Moreover, microsaccade rate has been shown to respond to odd stimuli in oddball tasks and can thus be considered as useful when investigating decision-making with rare targets^22,23^ or with visuospatial cues^44^. A sudden change in brightness, as well as a key press did not alter microsaccade rate for the stimulus-locked data (Fig. 5B,C). Decision making has been investigated using pupil dilation and microsaccade rates before; still, often microsaccade rate has been given as an additional proof for the validness of fMRI results, but has not been investigated more closely^18,20,21,45^. To our knowledge, pupil dilation and microsaccade rate in decision making have not been purposefully investigated before with physically identical targets and distractors, which only differed regarding their task-induced meaning. Our data imply that changes in microsaccade rate, which have previously been attributed to processes of decision, might at least partially be due to motor execution^20,45^.

Investigating a possible connection between microsaccade rates and pupil dilation by aligning pupil diameter to microsaccades did not reveal a clear coupling of microsaccades and a specific pupil response (Supplementary Fig. S4). We therefore assume, that a link between microsaccades and pupil dilation should be reflected in the overall microsaccade rate rather than in unique microsaccade events. When comparing microsaccade rates with pupil diameter courses, we found significant differences between target and distractor conditions for pupil diameter, but not microsaccade rate prior and during the key press. When response-locking pupil courses (Fig. 4A) and microsaccade rates (Fig. 6A), a clear indicative role for the timing of the key press became evident, which was also suggested in previous research^33,43,46^. Thus, microsaccades and pupil dilation both indicated an upcoming key press (Figs 4A and 6A). However, as assumed, the outcome of the decision could be predicted on the basis of pupil diameter (Fig. 4B), but not on the basis of microsaccade rate (Fig. 6B). Still, response-locked pupil dilation and microsaccade rates show corresponding results after choice, although there is a considerable lag and much smaller effect size for microsaccade rate compared to pupil dilation. This effect is in accordance with recent data investigating the predictive potential of LC- and SC-activity in a decision task^18^ and highlights the role of LC or a close correlate of LC but not SC for decision making. As pupil diameter and microsaccade rate covary for the key press, we argue that the joint investigation of both variables may help dissociating effects in pupil diameter from interfering factors.

## Methods

### Participants

30 students of Ulm University, including author one and author two participated in the study (*M*Age = 25.13; 16 female). Subjects took part on a voluntary basis after written informed consent was obtained; all subjects had normal or corrected to normal vision. The experiment was approved by the Ethical Board of Ulm University. All methods were performed in accordance with relevant guidelines and regulations. All subjects gave informed consent for taking part in the experiment.

### Task

The experiment was presented on a 27-inch screen (resolution set to 1920 × 1080 px, 144 Hz), located in a viewing distance of 60 cm to eye position. The participants’ task was to type words by gaze throughout the experiment. Participants were presented with a central quadractic shape of 4.5 degree visual angle edge length in addition to a similar colored shape of the same size at an eccentricity of 4.5 degree visual angle either upwards, downwards, leftwards or rightwards to the central object. Subjects were instructed to first fixate the peripheral object and then to saccade to the central shape. If the outer shape had been fixated before, a letter appeared in the central shape. The letter was highlighted with a dark blue or red frame around the shape after 3 s. Whenever subjects did not maintain their gaze position for the full 3 s within the central shape, the trial restarted with another either correct or incorrect letter to prevent a possible gaze-position-related confounding in the pupillary signal. At the upper rim of the screen, one out of 16 neutral words with four letters was constantly presented. Hereby, typed letters were written in black whereas not yet entered letters were displayed in gray. The letters presented in the middle of the screen either matched (=targets) the next letter of the word displayed above or not (=distractors) and were of equal probability to appear. When a target was selected, the respective letter in the target word turned black, hereby the next relevant letter was indicated.

In half of trials, a sinewave tone of 440 Hz was presented for 200 ms when saccading to the central shape. This tone prompted subjects to press a key indicating whether the displayed letter was correct (right arrow key) or not (left arrow key). Whether a key press had to be performed in response to the tone (subjects 1–15) or in response to no tone (subjects 16–30) was balanced across subjects. Moreover, background brightness was varied, i.e. in half of trials, illuminance at eye position was kept constant (60 lx), whereas the background turned dark in the other half of trials, leading to an illuminance of 20 lx at eye position, when saccading to the center. All conditions were presented in full random order. Once the full word was completed, the next randomly chosen word out of the still remaining words was displayed in gray until all words had been typed and the experiment ended. Otherwise, the experiment ended after 25 minutes.

### Preprocessing gaze data

Gaze was tracked at 500 Hz using a SMI iView X HiSpeed 1250 eye tracker in a brightness controlled laboratory after a nine point calibration and four point validation provided by the manufacturer was performed (www.smivision.com). Trials with a total duration of blinks longer than 200 ms were excluded from analyses. Blinks shorter than 200 ms were removed from the pupillary data using an algorithm that uses the physiological limits of changes in pupil diameter as a threshold for blink detection and then interpolates these blinks^47^, which we adapted to the 500 Hz sample rate. In a comparable experimental design^37^, it was suggested to use the middle of the incoming saccade to a central object as baseline. We chose ten data point equaling 20 ms oriented at the middle between activation box and the central box as approximation (as saccades of this length should take 40 ms)^48^. Baseline was subtracted from every following data point for the subsequent 3 s. However, for predicting key presses (illustrated in Fig. 4), baseline was set to one second before key press, aiming to control for the variance added to the pupillary signal by the key press (see Fig. 2A). Response-locked pupil diameter showed no significant difference (*t*(29) = −1.07, *p* = 0.29) at baseline between target and distractor conditions (target: *M* = 3.81, *SD* = 0.48; distractor: *M* = 3.82, *SD* = 0.48). Signal trends may affect the overall results of pupillometric investigations if significant changes to the baseline are interpreted as consequence of experimental manipulations, while they originate in an overall signal trend. We compared experimental conditions that were presented in randomized order to preclude this problem. Moreover, no overall increasing or decreasing signal trend was found for pupil diameter when comparing the average second minute (*M* = 3.68, *SD* = 0.49) with the average second last minute (*M* = 3.60, *SD* = 0.45) of each participant in the experiment with a paired samples t-test (*t*(29) = 1.54, *p* = 0.134).

Gaze data were classified into saccades, blinks, and fixations using SMI event detector with a minimum fixation duration set to 22 ms. For fixations, microsaccades were detected using the algorithm by Engbert and Kliegl^43^, with recently suggested improvements^49–51^ (Supplementary Fig. S5 for further details on the algorithm). Thresholds for velocity based microsaccade detection were determined only for fixations in the screen center. Microsaccades were accumulated in a moving window of 200 ms. This accumulated number of microsaccades was then divided by the accumulated time spent fixating during the same 200 ms.

For further analysis, pupil and microsaccade data were subsequently downsampled to 50 Hz. Data were then aggregated to form individual mean dynamics for each subject in each condition. These were subsequently aggregated to grand means and confidence intervals. Functional confidence intervals were chosen as main statistical visualization, given that they take the full temporal dynamics of the signals into account and prevent cherry-picking of specific intervals, which might result in an inflation of false positive results^4,52^. As multiple tests are conducted, the multiple comparison problem arises. Given the large number of tests performed here, a False Discovery Rate correction was chosen, which is applied in a wide variety of fields and has also been used before in pupillometric investigations^9^. Therefore, as Einhäuser *et al*.^9^, we applied a FDR correction to account for the problem of multiple testing, using the Benjamini-Hochberg method^53^. Signal courses are based on grand means composed of individual subject-average courses, based on a total of *n* = 4412 trials.

### Modeling

In order to estimate the effects of the different experimental conditions on pupil diameter over time, a linear mixed model (LMM) was calculated. A LMM was chosen to account for the nested structure of data, i.e. trials are nested in subjects and in conditions^54^. The model depicted in Table 1 was determined via stepwise AIC-based backward selection. The model depicted here for all time points was determined on the average pupil diameter for each trial in the interval between 0.5 s and 1.5 s after letter onset. Analyzes were performed using the statistical software R with lme4, lmerTest and MuMin packages^54–56^. Furthermore, a functional LMM was calculated for the factors brightness, choice, and key press, as well as their interactions (Fig. 3. In order to perform tests, degrees of freedom had to be estimated using Satterthwaite’s approximation, *α* = 0.05 was FDR-corrected^56^.

## Electronic supplementary material

Supplementary Information: Pupil dilation but not microsaccade rate robustly reveals decision formation

## Data Availability

The datasets generated during and/or analyzed during the current study are available in the Open Science Framework repository, https://osf.io/kjrze/.
