# Supplementary material for: Pupil dilation but not microsaccade rate robustly reveals decision formation
Source: Sci Rep. 2018 Sep 3;8:13165. doi: 10.1038/s41598-018-31551-x (PMC6120888; doi:10.1038/s41598-018-31551-x)
Supplement: Supplementary file 1 — Supplementary Information: Pupil dilation but not microsaccade rate robustly reveals decision formation [file 41598_2018_31551_MOESM1_ESM.pdf]

Supplementary Information: Pupil dilation but not microsaccade rate robustly reveals decision formation

Christoph Strauch[1], Lukas Greiter[1], and Anke Huckauf[1]

[1]Ulm University, General Psychology, Ulm, 89081, Germany

SI Results

In Figure S1, stimulus-locked pupil responses for all subjects for target/distractor in constant/reduced brightness and Go/NoGo conditions are illustrated in the same way as in Figure 2B.

In Figure S2, pupil dilation is illustrated as a function of the tone being played. The tone prompted the subject either to press a key (subjects 1-15) or not to press a key (subjects 16-30). In Figure S2A, pupil diameter changes for trials with tone and without tone are visualized. Figure S2B depicts the average difference between trials with tone and without tone. A significantly larger pupil dilation for trials with tone compared to trials without tone between 0.6 s and 0.9 s after letter onset was observed. Figure S2C depicts the difference between target and distractor trials after subtracting trials without tone from trials with tone. No significant interaction between choice and tone was monitored. In Figure S3, the effect of the tone on microsaccade occurrence is illustrated. Figure S3A depicts microsaccade rates for trials with tone compared to trials without tone. It appears that the general microsaccade response is shifted in trials with tone compared to trials without tone. However, this difference does not lead to a significantly higher microsaccade rate when being analyzed functionally (Fig. S3B). No interaction between tone and choice was found (Figure S3C). The shift in microsaccade rate between trials with and without tone cannot be explained by differential reaction times, as these did not differ significantly (both $M = 1.07$ s; $U = 84.0$, $p = 0.98$).

In Figure S4, average pupil diameter is depicted aligned to microsaccades that occurred during the trials. Depending on the experimental condition and the respective microsaccade rate over time, as well as on differential pupil responses for the experimental conditions, microsaccade-aligned pupil responses descriptively differ. Overall, the increasing trend that was monitored for pupil data within trials is reflected in this data, however, no specific pattern was found prior to or following microsaccades. Therefore, any link between microsaccade and pupil dilation should be reflected in the overall microsaccade rate rather than in unique microsaccade events.

SI Methods

For an example of a sequence of trials, see 'Example trials.gif' at `https://osf.io/kjrze/`. The microsaccade detection algorithm is illustrated as a flowchart in Figure S5 (see 'Preprocessing gaze data' in the 'Methods' section for references).

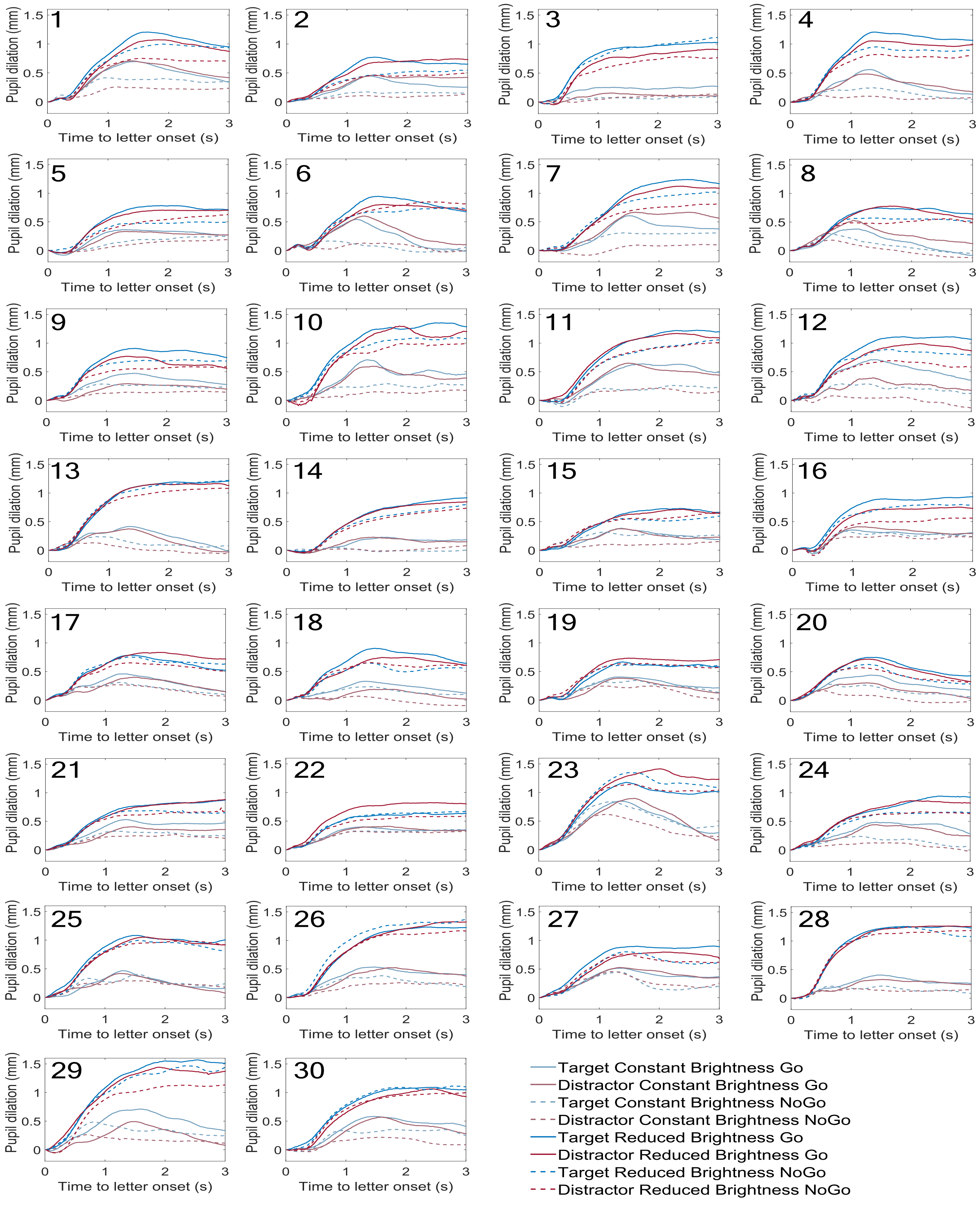

Figure S1. Stimulus-locked pupil data for all subjects, refer to Figure 2B of the main paper for grand average courses. Target trials are depicted in blue (distractor: red) with dark blue/red for trials with reduced brightness and lighter blue for trials with constant brightness. Dashed lines mark NoGo-trials (Go-trials: solid lines). Subject number is indicated in the upper left corner of each graph.

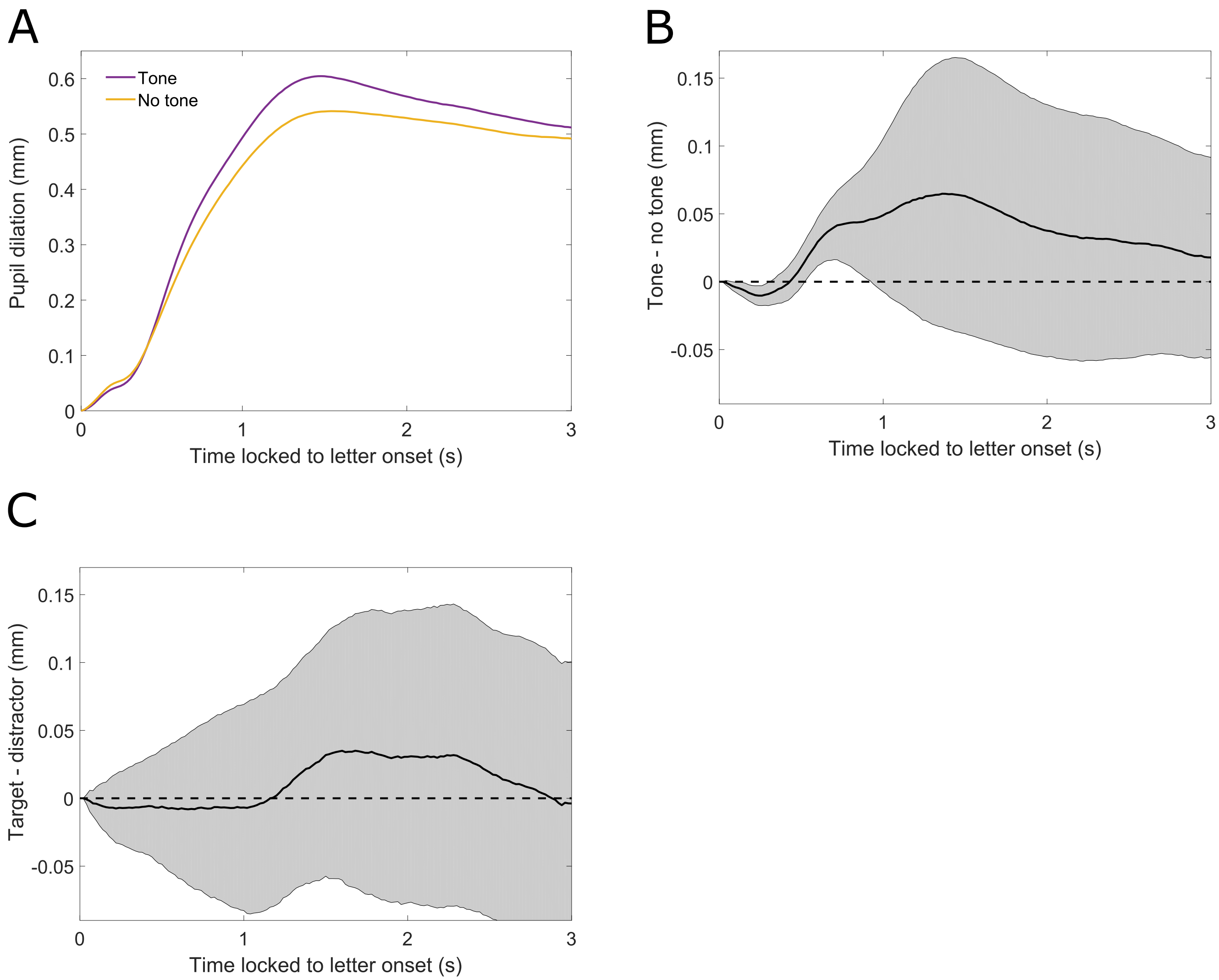

Figure S2. Stimulus-locked pupil data. (A) average pupil size changes for trials with tone and without tone. (B) functional difference between trials with and without tone (solid black line) with functional confidence intervals (shaded grey area). If zero is not contained in the CI, changes are significantly different to FDR-corrected $\alpha = 0.05$. (C) functional difference between target and distractor after subtracting trials without tone from trials with tone.

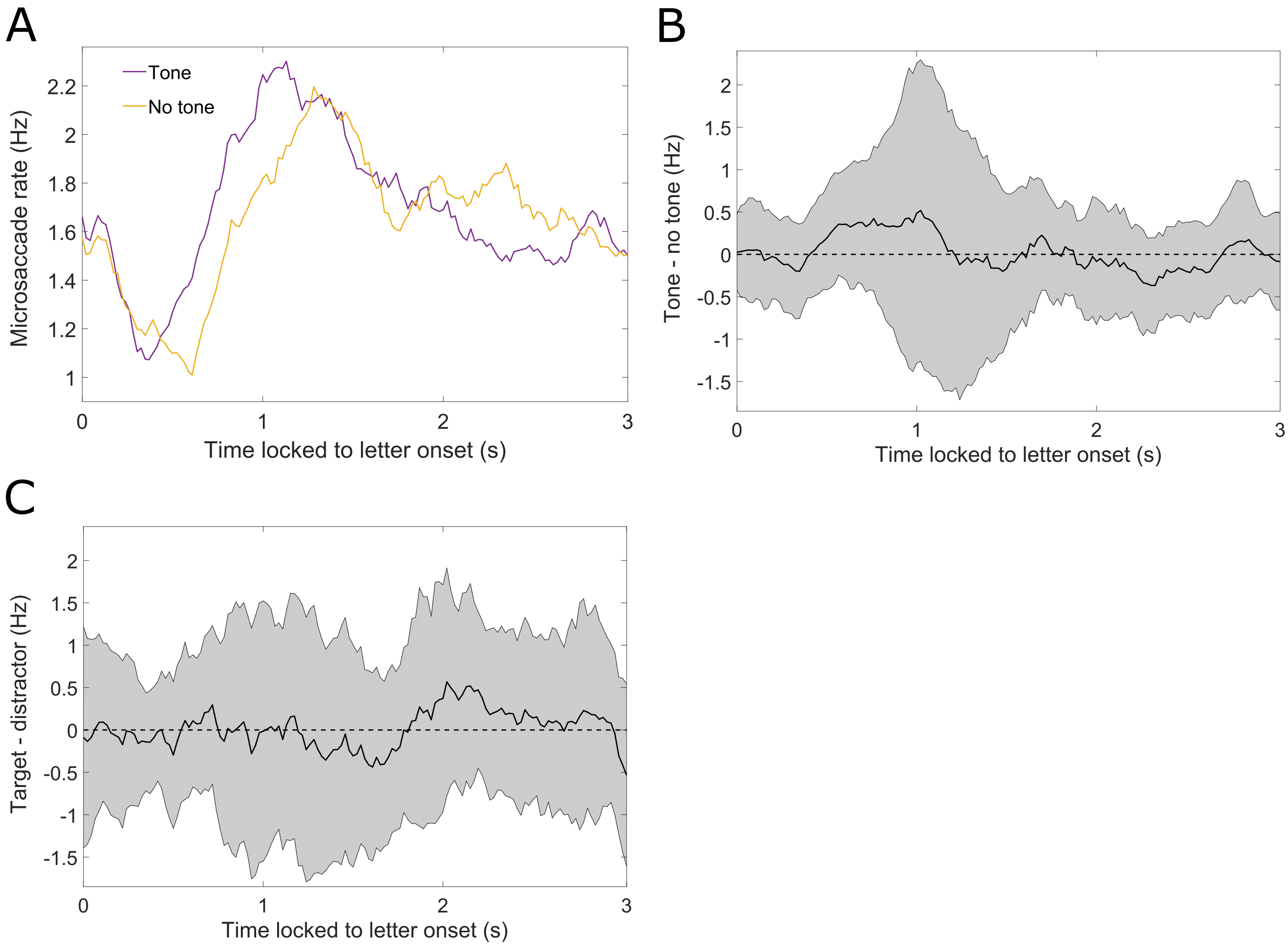

Figure S3. Stimulus-locked microsaccade rates. (A) average microsaccade rate for trials with tone and without tone. (B) functional difference between trials with and without tone (solid black line) with functional confidence intervals (shaded grey area). If zero is not contained in the CI, changes are significantly different to FDR-corrected $\alpha = 0.05$. (C) functional difference between target and distractor after subtracting trials without tone from trials with tone.

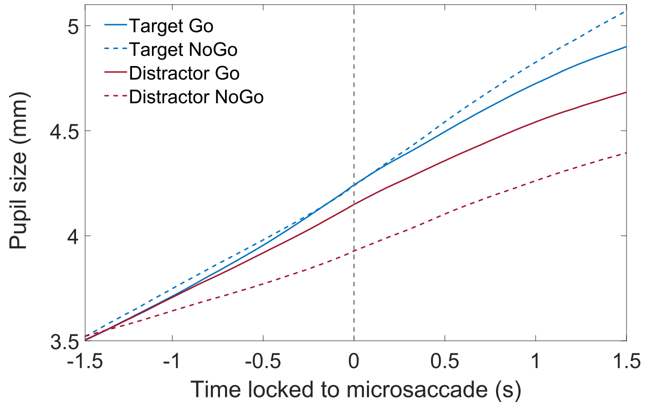

Figure S4. Pupil size aligned to microsaccades that occurred during trials.

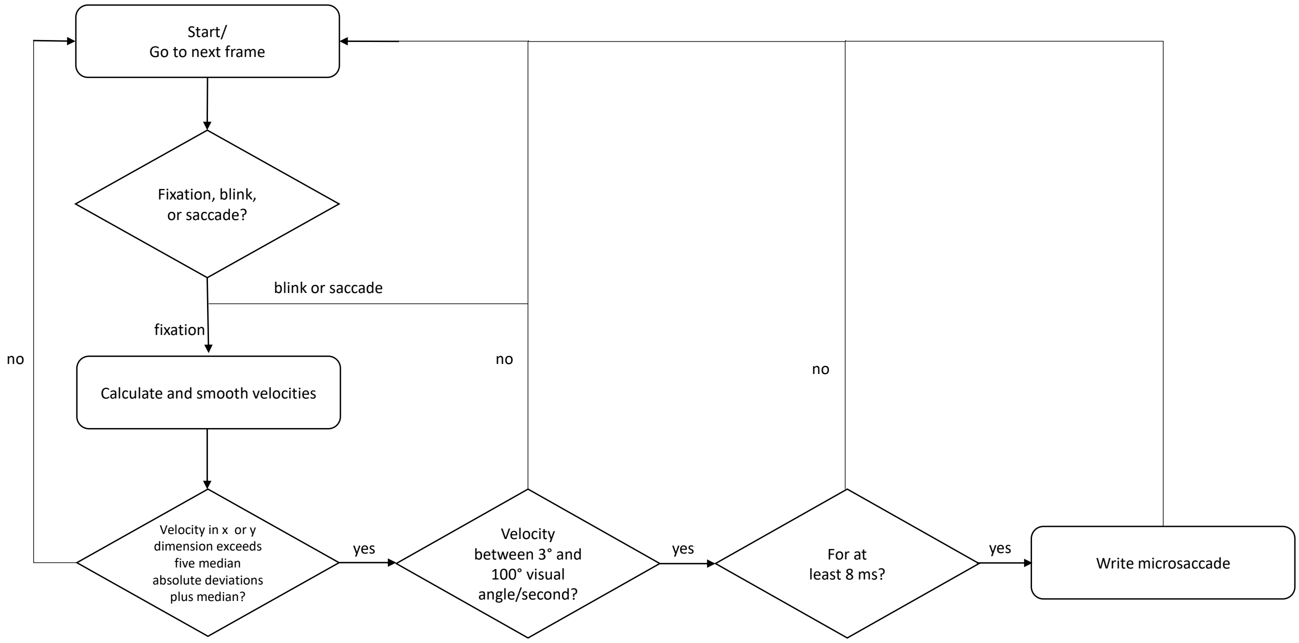

Figure S5. Flowchart for the microsaccade detection algorithm.
